# Clinical and neuroanatomical characterization of the semantic behavioral variant of frontotemporal dementia in a multicenter Italian cohort

**DOI:** 10.1007/s00415-024-12338-9

**Published:** 2024-04-10

**Authors:** Alma Ghirelli, Edoardo Gioele Spinelli, Elisa Canu, Silvia Basaia, Veronica Castelnovo, Giordano Cecchetti, Elisa Sibilla, Teuta Domi, Giuseppe Magnani, Francesca Caso, Paola Caroppo, Sara Prioni, Cristina Villa, Giacomina Rossi, Lucio Tremolizzo, Ildebrando Appollonio, Federico Verde, Nicola Ticozzi, Vincenzo Silani, Massimo Filippi, Federica Agosta

**Affiliations:** 1 Neuroimaging Research Unit, Division of Neuroscience, IRCCS San Raffaele Scientific Institute, Via Olgettina 60, 20132 Milan, Italy; 2 Vita-Salute San Raffaele University, Milan, Italy; 3 Neurology Unit, IRCCS San Raffaele Scientific Institute, Milan, Italy; 4 Experimental Neuropathology Unit, Division of Neuroscience, IRCCS San Raffaele Scientific Institute, Milan, Italy; 5 Unit of Neurology 5-Neuropathology, Fondazione IRCCS Istituto Neurologico Carlo Besta, Milan, Italy; 6 Neurology Unit, “San Gerardo” Hospital and University of Milano-Bicocca, Monza, Italy; 7 Department of Neurology and Laboratory of Neuroscience, IRCCS Istituto Auxologico Italiano, Milan, Italy; 8 “Dino Ferrari” Center, Department of Pathophysiology and Transplantation, Università Degli Studi di Milano, Milan, Italy; 9 Neurophysiology Service, IRCSS San Raffaele Scientific Institute, Milan, Italy; 10 Neurorehabilitation Unit, IRCCS San Raffaele Scientific Institute, Milan, Italy

**Keywords:** sbvFTD, FTD, MRI, Voxel-based morphometry, rtvFTD

## Abstract

**Background:**

Semantic behavioral variant frontotemporal dementia (sbvFTD) is a neurodegenerative condition presenting with specific behavioral and semantic derangements and predominant atrophy of the right anterior temporal lobe (ATL). The objective was to evaluate clinical, neuropsychological, neuroimaging, and genetic features of an Italian sbvFTD cohort, defined according to recently proposed guidelines, compared to semantic variant primary progressive aphasia (svPPA) and behavioral variant FTD (bvFTD) patients.

**Methods:**

Fifteen sbvFTD, sixty-three bvFTD, and twenty-five svPPA patients and forty controls were enrolled. Patients underwent clinical, cognitive evaluations, and brain MRI. Symptoms of bvFTD patients between onset and first visit were retrospectively recorded and classified as early and late. Grey matter atrophy was investigated using voxel-based morphometry.

**Results:**

sbvFTD experienced early criteria-specific symptoms: world, object and person-specific semantic loss (67%), complex compulsions and rigid thought (60%). Sequentially, more behavioral symptoms emerged (apathy/inertia, loss of empathy) along with non-criteria-specific symptoms (anxiety, suspiciousness). sbvFTD showed sparing of attentive/executive functions, especially compared to bvFTD and better language functions compared to svPPA. All sbvFTD patients failed at the famous face recognition test and more than 80% failed in understanding written metaphors and humor. At MRI, sbvFTD had predominant right ATL atrophy, almost specular to svPPA. Three sbvFTD patients presented pathogenic genetic variants.

**Conclusion:**

We replicated the application of sbvFTD diagnostic guidelines in an independent Italian cohort, demonstrating that the presence of person-specific semantic knowledge loss and mental rigidity, along with preserved executive functions and a predominant right ATL atrophy with sparing of frontal lobes, should prompt a diagnosis of sbvFTD.

**Supplementary Information:**

The online version contains supplementary material available at 10.1007/s00415-024-12338-9.

## Introduction

Frontotemporal dementia (FTD) is a broad term that includes various syndromes characterized by the progressive loss of inhibitory circuits and language pathways and gradual involution of frontotemporal cortex. In addition to the three canonical clinical presentations of FTD described by currently recognized criteria (i.e., the behavioral variant of FTD [bvFTD] [1], and the semantic [svPPA] and non-fluent/agrammatic [nfvPPA] variants of primary progressive aphasia) [2], a clinico-radiological syndrome characterized by distinctive behavioral and semantic derangements associated with predominant atrophy of the right anterior temporal lobe (ATL) has been recognized as a separate diagnostic entity by some studies [3–5]. Given that ATL degeneration is typically associated with transactive-response DNA-binding protein 43 type C (TDP type C) pathology [6] and that this syndrome presents with a complex and unique constellation of symptoms, early diagnosis might facilitate the initiation of a tailored rehabilitation program and timely enrollment into treatment trials.

Diagnostic clinical guidelines to encapsulate this syndrome have been recently proposed [7]. These tentative criteria suggest the use of the term semantic behavioral FTD (sbvFTD), providing three levels of diagnostic certainty (clinical, imaging-supported, and definite pathology), similarly to other FTD syndromes [1, 2]. The core clinical features identified by these guidelines are: (a) loss of empathy—usually, occurring early in the disease course; (b) difficulty in naming and identifying known people; (c) complex compulsions or rigid though process. For an imaging-supported diagnosis of sbvFTD, predominant right ATL volume loss with relative sparing of left ATL and bilateral frontal cortices should be also demonstrated.

These guidelines [7] have been the first attempt to categorize this complex clinical variant of FTD. As such, they need further validation in external cohorts to clarify their sensitivity in culturally diverse populations. Furthermore, there is the need to define instruments to differentiate sbvFTD from the two close variants bvFTD and svPPA at a clinical, neuropsychological, and neuroimaging level. Therefore, the aims of our study were (1) to apply Younes diagnostic guidelines [7] to an Italian FTD cohort, (2) to evaluate the presence of additional clinical features, (3) to evaluate neuropsychological instruments that could pick peculiar features of this variant, and (4) to evaluate clinical and/or radiological overlapping elements with bvFTD and svPPA. The final aim was to test applicability of Younes diagnostic guidelines [7] in an independent cohort and possibly highlight critical features or novel elements that should be considered in the evaluation of sbvFTD patients.

## Methods

### Participants

**Fig. 1:**
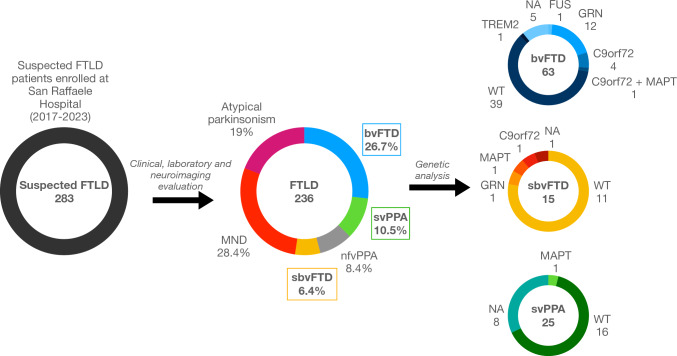
Sample selection and study design. Two hundred eighty-three patients with a suspected diagnosis of FTLD-spectrum disease were enrolled at San Raffaele Hospital between 2017 and 2023. Of these, 236 were confirmed for an FTLD-related disorder. Fifteen patients (6.4%) had sbvFTD. The remaining patients had bvFTD (*n* = 63, 26.7%), svPPA (*n* = 25, 10.5%), nfvPPA (*n* = 21, 8.4%), MND (*n* = 67, 28.4%), atypical parkinsonism (*n* = 45, 19%). For the purposes of this study, we included only patients with a diagnosis of sbvFTD, bvFTD or svPPA, for a total number of 103 subjects. Detected genetic mutations are reported in the last pie chart. *bvFTD* behavioral variant frontotemporal dementia, *C9orf72* chromosome 9 open reading frame 72, *FTLD* frontotemporal lobar degeneration, *FUS* fused in sarcoma, *GRN* progranulin, *MAPT* microtubule associated protein tau, *MND* motor neuron disease, *sbvFTD* semantic behavioral variant frontotemporal dementia, *svPPA* semantic variant primary progressive aphasia, *TREM2* triggering receptor expressed on myeloid cells 2

Figure 1 outlines the screening process. A total of 283 patients with a suspected diagnosis of FTLD-related disorders were enrolled in 4 referral clinics in Lombardy, Italy, and referred to San Raffaele Hospital in Milan between June 2017 and January 2023 to undergo brain MRI on a 3 T scanner. Of these, 236 were confirmed for an FTLD-related disorder. By applying Younes diagnostic guidelines [7], based on review of clinical and routine MRI data, we identified 15 patients (6.4%) with an imaging-supported diagnosis of sbvFTD [7]. The remaining patients presented with a diagnosis of bvFTD (*n* = 63, 26.7%) [1], svPPA (*n* = 25, 10.5%) [2], nfvPPA (*n* = 21, 8.8%) [2], motor neuron disease (*n* = 67, 28.4%) [8–10] or atypical parkinsonism (progressive supranuclear palsy or corticobasal syndrome) (*n* = 45, 19%) [11, 12]. For this study, we included only patients with a diagnosis of sbvFTD, bvFTD or svPPA, for a total number of 103 subjects. Forty healthy controls matched with patients by age, sex, and education were also included. Exclusion criteria for patients with suspected FTLD-related disorders included: evidence of Alzheimer’s pathology at lumbar puncture, absence of signs of neurodegeneration at MRI/18-fluorodeoxyglucose PET, or evidence of a high vascular load at MRI that prompted a diagnosis of vascular dementia. All healthy controls fulfilled the following criteria: normal neurologic assessment, Mini-Mental State Examination (MMSE) score ≥ 28, and no family history of neurodegenerative diseases. Exclusion criteria for all participants were medical illnesses or substance abuse that could interfere with cognitive functioning; any (other) major systemic, psychiatric, or neurologic illnesses.

### Genetic analysis

Blood samples were collected from 89 out of 103 patients and genomic DNA was obtained and processed in each of the recruiting centers. Details on genetic analysis are discussed in the Supplementary Material.

### sbvFTD symptomatic classification

All participants were evaluated by a behavioral neurologist and a neuropsychologist. Patients’ clinical history was reviewed based on clinical charts, with corroboration from the caregiver/informant. Symptoms developed between disease onset and the first visit in our center were retrospectively recorded. A chronological history of how symptoms evolved was performed, documenting each sbvFTD patient’s first three symptoms. Symptoms were then classified as early (first three symptoms reported) or later emerging (symptoms developed subsequently). Symptoms were classified according to the taxonomy elaborated by Younes et al. [7] into:

- Loss of empathy
- Words and object semantic loss
- Person-specific semantic knowledge loss
- Complex compulsions and rigid thought process
- Simple repetitive behaviors, hoarding or obsessions
- Apathy/inertia
- Disinhibition
- Lack of judgement and dysexecutive
- Episodic memory loss
- Hyperorality or dietary changes
- Motor neuron disease signs
- Other symptoms (visuospatial difficulties, declines hygiene, loss of sexual desire, increased or decreased eating, weight gain/loss, hypersomnia, and insomnia)

Furthermore, given the high presence of behavioral symptoms that did not fit into any of the available categories, we introduced a new domain, referred as “Extra-criteria”. Patients included in this category presented with anxiety, suspiciousness, agitation or irritability, which were not explicitly mentioned in the description provided by Younes et al. [7], not even among the “Other symptoms” category. The definition of the “Extra-criteria” category in our report aims to highlight behavioral nuances that are strictly correlated with mental rigidity and obsessions but cannot be formally classified as such.

### Neuropsychological evaluation

Neuropsychological assessments were performed by experienced neuropsychologists, unaware of MRI results. A detailed outline of the neuropsychological evaluation is described in the Supplementary Materials. Due to the suspicion of ‘right-temporal’ suggestive symptoms as reported in the sole anamnesis by the caregiver/informant (i.e., prosopagnosia, mental rigidity), most of the sbvFTD (80%) and few bvFTD (11%) cases were also addressed by the neurologist to be administered with the Right Hemisphere Language Battery (Batteria sul Linguaggio dell’Emisfero Destro-BLED) [13] for specifically assessing the pragmatic language dysfunction in these patients, the Cognitive Estimation Task (CET), [14] and with both the Benton face recognition test [15] and the famous face naming test [16] for assessing prosopagnosia.

For each group, we reported only the cognitive scores of tests performed at least by 50% of subjects. Supplementary Table 1 reports the exact percentage of patients that underwent a specific test in each group.

### MRI study

Patients and healthy controls underwent brain MRI on a 3 T scanner (Philips Medical Systems) at San Raffaele Hospital between 2017 and 2023. Full details of the MRI acquisition protocol are reported in Supplementary Table 2. Voxel-based morphometry (VBM) was performed using SPM12 (http://www.fil.ion.ucl.ac.uk/spm/) and Diffeomorphic Anatomical Registration Exponentiated Lie Algebra (DARTEL) registration method [17] was utilized to investigate gray matter (GM) volume alterations, as described previously [18].

### Statistical analysis

Clinical, cognitive, and neuroimaging data were compared among groups. Details on the statistical analysis are reported in the Supplementary Material.

## Results

### Clinical and sociodemographic features

Sociodemographic and clinical characteristics of groups are reported in Table 1. Groups were comparable in terms of age at MRI, sex, handedness, and disease duration. Age of onset was similar among groups of patients, with an average of 60.7 years (SD 8.1). The average time elapsed between symptoms onset and MRI was 44 months (SD 32.3). Education was significantly higher in svPPA compared to bvFTD. As for disease severity, bvFTD patients scored higher compared to other groups at CDR-SB. Of note, 5 out of 15 patients (33%) classified as sbvFTD [7] formally met Rascovsky criteria [1] for bvFTD as well.

**Table 1:** Main sociodemographic and clinical characteristics of included subjects

	HC	sbvFTD	bvFTD	svPPA	*p*
*N*	40	15	63	25	–
Age at MRI	62 ± 9 [41–77]	61.8 ± 9 [48–77]	65.9 ± 7.1 [46–79]	66.4 ± 9.7 [42–81]	0.077
Females (%)	22 (55%)	5 (33.3%)	26 (41.3%)	13 (52%)	0.340
Handedness (right/left)	39/1	14/1	62/1	24/1	0.928
Age at onset	–	58.9 ± 8.1 [46–75]	61.6 ± 6.9 [43–77]	60.1 ± 10.6 [36–76]	0.525
Education (years)	11.8 ± 3.6 [5–18]	10.8 ± 4.1 [5–21]	10 ± 3.1[3–18]	12.5 ± 4.3 [5–18]^§^	**0.006**
Time onset to diagnosis (months)	–	32 ± 14.9 [9–60]	32.6 ± 29.5 [1–120]	33 ± 15.6 [13–63]	0.994
Time onset to MRI (months)	–	37.6 ± 22.7 [13–103]	40.5 ± 24.5 [4–121]	60.5 ± 50.8 [13–242]	0.059
Pathogenic genetic variants	–	C9orf72=1/GRN=1/MAPT=1	C9orf72=4/GRN=12/C9orf72+MAPT=1/FUS=1/TREM2=1	MAPT=1	–
CDR	–	0.7 ± 0.6 [0–2]	1.3 ± 0.9 [0–3]	1 ± 0.8 [0–3]	0.065
CDR-SB	–	4.5 ± 4.2 [1–14]	7.8 ± 5.1 [1–18]^$^^	5.1 ± 4.9 [0.5–16]	**0.038**
CDR® + NACC FTLD	–	5.6 ± 4.5 [2.5–18]	10.1 ± 6 [1–23]	7.9 ± 6.3 [1–21]	0.057

Values denote means ± standard deviations [range] or frequencies (%)

Bold is for significant values

*bvFTD* behavioral variant FTD, *CDR* clinical dementia rating, *CDR-SB* clinical dementia rating sum of boxes, *CDR* + *NACC FTLD* clinical dementia rating + National Alzheimer’s Coordinating Center Frontotemporal Lobar Degeneration, *HC* healthy controls, *sbvFTD* semantic behavioral variant FTD, *svPPA* semantic variant primary progressive aphasia

^§^Statistically different from bvFTD

^$^Statistically different from sbvFTD

^^^Statistically different from svPPA

### Genetic findings

As outlined in Fig. 1**,** out of 14 sbvFTD patients for whom genetic analysis was available, 3 (21%) presented pathogenic variants, respectively, in the *C9orf72* (*N* = 1), *GRN* (*N* = 1), and *MAPT* gene (*N* = 1). For what concerns bvFTD, out of 58 patients with genetic analysis available, a total of 19 patients (33%) presented pathogenic variants: *C9orf72* (*N* = 4), *GRN* (*N* = 12), *TREM2* (*N* = 1), *FUS* gene (*N* = 1), or in both *C9orf72* and *MAPT* genes (double mutation, *N* = 1). Only 1 svPPA patient out of 17 with genetic analysis available (6%) carried a known pathogenic mutation in the *MAPT* gene. Supplementary Table 3 reports the details of each clinical variant identified in our cohort.

### Symptom unfolding in sbvFTD

Supplementary Table 4 summarizes the first three symptoms developed by each sbvFTD patient as reported by caregivers and/or patients themselves. The most frequent earliest complaints experienced by patients included criteria-specific symptoms: word and object semantic loss (67%), person-specific semantic knowledge loss (67%), and complex compulsions and rigid though process (60%). Another 47% of patients reported experiencing apathy/inertia, 27% presented simple repetitive behaviors, hoarding or obsessions, as well as loss of empathy. Only 20% of patients described episodic memory loss and, eventually, 13% of patients reported hyperorality or dietary changes. Also, 27% of patients experienced early “extra-criteria” symptoms (i.e., non-criteria-specific). The main feature experienced by those patients was anxiety, with the tendency of suspiciousness and in general easy irritability. No patient had early lack of judgement and dysexecutive symptoms or disinhibition.

**Fig. 2:**
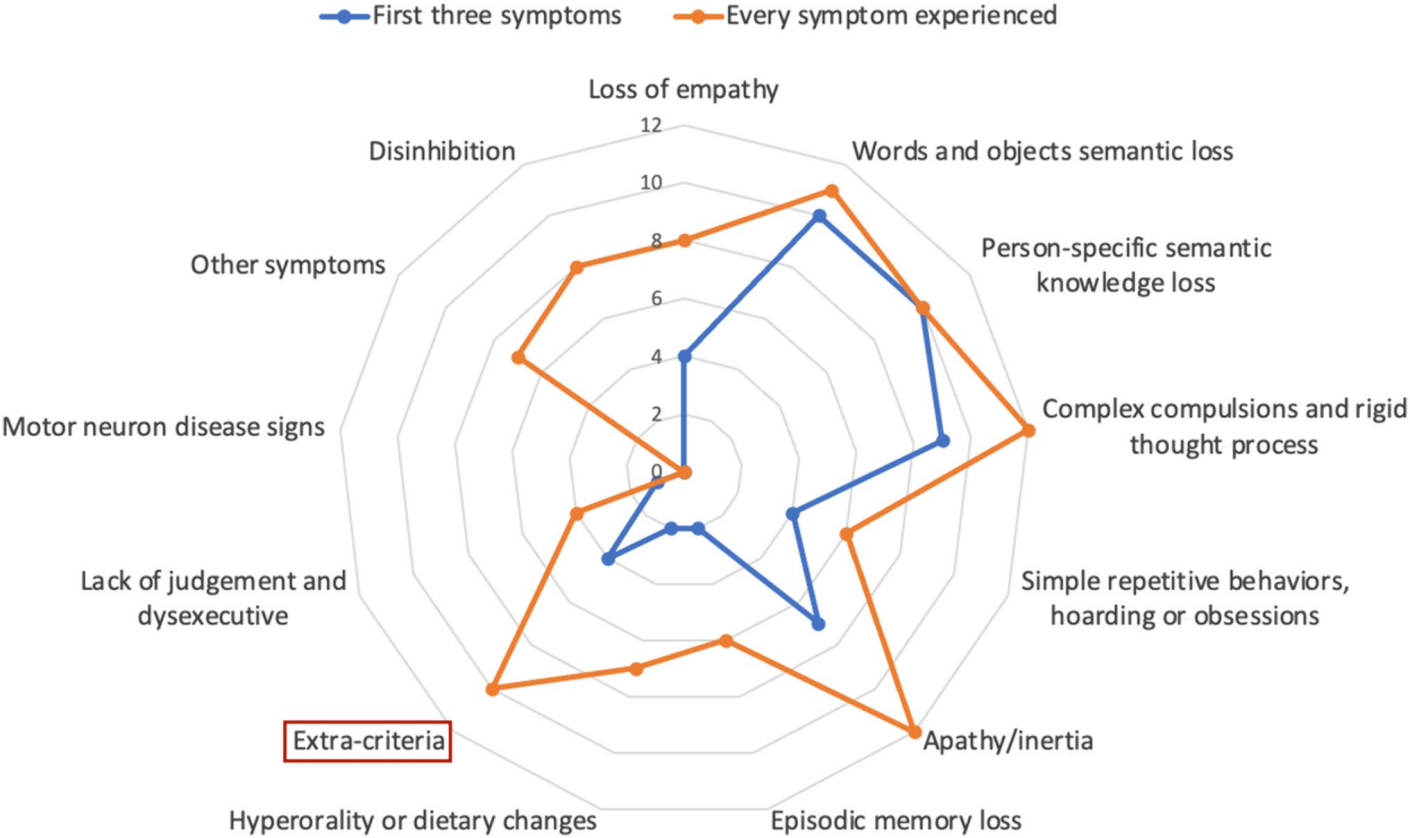
Symptoms developed by semantic behavioral FTD (sbvFTD) patients. Spider chart depicting the number of sbvFTD patients affected by a given symptom. Blue line represents the first three symptoms reported by caregivers/patients; orange line represents all symptoms reported from disease onset to the time of the visit. “Extra-criteria” symptoms = anxiety, suspiciousness, agitation, irritability or depression

As the disease progressed, the number of patients experiencing general behavioral symptoms as well as episodic memory loss increased. Indeed, at the time of the visit, recollecting symptoms from disease onset up to referral to our center, up to 80% of patients had developed apathy/inertia, as well as complex compulsions and rigid though process, 73% words and objects semantic loss, 53% presented disinhibition and loss of empathy, 50% developed simple repetitive behaviors, hoarding and obsessions and episodic memory loss, 51% developed hyperorality or dietary changes, 27% developed lack of judgement or dysexecutive symptoms, while 47% developed other symptoms (all criteria-specific symptoms). The percentage of patients experiencing “extra-criteria” symptoms increased from 27 to 67%. No patient developed motor neuron disease signs. A visual representation of symptom unfolding is provided in Fig. 2.

To better characterize the type of symptoms referred, we asked caregivers to provide real-life examples. Patients complaining complex compulsions presented with restricted food preference (e.g., only ate bread and pasta; healthy food; pre-cooked food; sophisticated food; cold dishes and fruit), had fixed daily routine (i.e., must set up the table 3 h before diner, had to spend exactly 1 h a day at the bakery shop), had the tendency to compulsively look for a job after having lost it, could not let the phone battery go below 80%, or experienced psychiatric symptoms such as potomania or opiate abuse. As for person-specific semantic knowledge loss, the main symptoms reported were the inability to recognize either famous or familiar faces (colleagues, relatives) or recalling proper names. Among other “extra-criteria” behavioral derangements were the inability to understand jokes, anxiety, preoccupation, histrionic-like behavior, and irritability.

### Neuropsychological features

Table 2 describes the main neuropsychological features of our cohort. sbvFTD patients were more preserved in terms of verbal working memory and selective attention (attentive matrices) compared to both bvFTD and svPPA, limb ideomotor praxis (Goldenberg’s test) and affect discrimination (CATS, affect discrimination) compared to bvFTD, global cognition (MMSE) and naming compared to svPPA.

**Table 2:** Neuropsychological scores of patients and healthy controls

	HC	sbvFTD	bvFTD	svPPA	*p*
*N*	40	15	63	25	–
Global cognition					
MMSE	29.4 ± 0.9 [27–30]	26.2 ± 3.9 [18–30]*	22.4 ± 6.1 [6–30]*	19.1 ± 8.7 [5–30]*^$^	**< 0.001**
FAB	–	12.9 ± 3.1[5–17]	10.3 ± 4.6 [1–17]	11.9 ± 4 [3–17]	0.08
Memory					
RAVLT, immediate recall	48.5 ± 6.3 [39–60]	29.9 ± 11.5 [14–48]*	23.7 ± 9.7 [0–43]*	21.7 ± 11.8 [0–43]*	**< 0.001**
RAVLT, delayed recall	10.7 ± 2.5 [4–15]	4.6 ± 3.6 [0–12]*	2.8 ± 2.8 [0–9]*	2.6 ± 3.3 [0–10]*	**< 0.001**
Benson figure, recall	11.3 ± 2.6 [6–17]	6.5 ± 3.6 [0–12]*	4.2 ± 3.9 [0–16]*	5.9 ± 5.4 [0–16]*	**< 0.001**
Benson figure, recognition	1 ± 0 [1]	0.6 ± 0.5 [0–1]*	0.5 ± 0.5 [0–1]*	0.5 ± 0.5 [0–1]*	**< 0.001**
Digit span, forward	6.1 ± 1 [4–8]	5.4 ± 0.9 [3–7]	4.8 ± 1.5 [0–7]*	4.7 ± 1.1 [2–6]*	**< 0.001**
Spatial span, forward	5.5 ± 1.1 [4–7]	4.5 ± 1.6 [0–7]*	3.6 ± 1.5 [0–7]*	4.3 ± 1.2 [2–7]*	**< 0.001**
Visuospatial abilities					
Benson figure, copy	15.8 ± 0.8 [14–17]	15.4 ± 1 [13–17]	12.4 ± 4.2 [0–17]*^$^^	14.5 ± 2.3 [7–17]	**< 0.001**
CDT	–	6.7 ± 3.8 [0–10]	5.3 ± 3.7 [0–10]	4.2 ± 4.2 [0–10]	0.09
Executive functions					
Raven’s colored progressive matrices	32.4 ± 2.9 [23–36]	24.9 ± 7.6 [5–35]*	19.6 ± 8.7 [4–35]*	23 ± 10.1 [3–36]*	**< 0.001**
Digit span, backward	4.8 ± 1.3 [3–8]	4.29 ± 1 [2–6]	2.8 ± 1.5 [0–5]*^$^	2.8 ± 1.6 [0–5]*^$^	**< 0.001**
MCST, categories	4.4 ± 1.4 [1–6]	3.7 ± 2.3 [0–6]	2.1 ± 1.7 [0–6]*^$^^	4.5 ± 1.4 [2–6]	**< 0.001**
MCST, perseverations	3.7 ± 3.5 [0–12]	6 ± 7 [0–20]	14.4 ± 10.1 [0–44]*^^^	4.3 ± 4 [0–13]	**< 0.001**
TMT, A	32.5 ± 8 [17.5–52.4]	62.2 ± 32.6 [30–131]	106.6 ± 105.3 [25–600]*	103.1 ± 132 [16.8 – 637]*	**0.001**
TMT, B	89.72 ± 25 [46.7–171]	164.4 ± 54.5 [105 – 278]	232.6 ± 131.1 [65 – 660]*	179.9 ± 100 [79–436]*	**<0.001**
TMT, BA	56.7 ± 23.8 [19.9–139]	102.2 ± 37.7 [43–180]	163.4 ± 126.6 [14–617]*	125.6 ± 87.1 [26–340]	**<0.001**
Attentive matrices	53.8 ± 5.4 [39–60]	49.3 ± 6.3 [35–59]	37.5 ± 15.3 [4–60]*^$^	37.3 ± 15.9 [12–56]*^$^	**<0.001**
Language					
Token test	34.5 ± 1.4 [31–36]	29.6 ± 5 [19.5–35]	26.3 ± 8.4 [5–36]*	22.4 ± 12.1 [3–36]*	**<0.001**
CaGi, visual naming	–	32.1 ± 13 [0–46]	–	17.4 ± 13.4 [0–35]^**$**^	**0.02**
CaGi, single–word comprehension	–	43.4 ± 5 [31–48]	–	38.3 ± 10.9 [10–48]	0.14
Phonemic fluency	37.2 ± 9.2 [21–59]	18.01 ± 6.6 [7–33]*	14.8 ± 10.6 [0–47]*	15.4 ± 11.1 [0–31]*	**<0.001**
Semantic fluency	47 ± 9.8 [27–70]	23.2 ± 11.2 [0–38]*	19.6 ± 9.8 [1–42]*	9.6 ± 7.8 [0–23]*^$§^	**<0.001**
Pyramids and Palm Trees test	–	39.1 ± 6.3 [27–47]	–	37.6 ± 6.8 [27–51]	0.33
Praxis					
Orofacial apraxia, ideomotor	–	18.1 ± 2.3 [13–20]	17.9 ± 3 [7–20]	17 ± 4 [8–20]	0.61
Orofacial apraxia, ideational	–	14 ± 6.4 [5–20]	16.7 ± 4.2 [3–20]	10.1 ± 6.4 [0–20]^§^	**0.002**
Limb apraxia, ideational right	–	12.8 ± 6.1 [3–20]	16.65± 4.7 [4–20]	8.2 ± 7 [0–20]^§^	**<0.001**
Limb apraxia, ideational left	–	12.8 ± 6.9 [3–20]	17 ± 4 [5–20]	8.5 ± 6.4 [0–20]^§^	**<0.001**
Limb apraxia, ideomotor right	–	19.4 ± 1.4 [15–20]	18.2 ± 3.4 [2–20]	19 ± 1.4 [15–20]	0.23
Limb apraxia, ideomotor left	–	19.8 ± 0.7 [18–20]	18.3 ± 3.5 [4–20]	18.6 ± 2.1 [11–20]	0.67
Goldenberg’s test, right	–	19.3 ± 1.1 [17–20]	15.9 ± 4.2 [0–20]^$^	16.9 ± 3.1 [10–20]	**0.02**
Goldenberg’s test, left	–	18.8 ± 1.7 [15–20]	15.8 ± 4.4 [0–20]^$^	18.2 ± 1.4 [14–20]^**§**^	**0.008**
Emotion and social cognition					
CET	–	19.2 ± 6.4 [10–34]	17.4 ± 7.2 [3–26]	–	0.62
Famous face recognition test	–	3.5 ± 8.1 [0–20]	–	–	NA
Benton face recognition test	–	37.2 ± 7.7 [23–44]	–	–	NA
SET					
SET, global score	–	9.1 ± 2.5 [5–13]	10 ± 3.5 [5–16]	10.5 ± 5.2 [2–18]	0.88
SET, intention attribution	–	2.8 ± 1.4 [0–5]	3.4 ± 1.6 [1–6]	3.5 ± 2 [0–6]	0.54
SET, causal interference	–	3.5 ± 1.8 [1.7–5.4]	3.6 ± 1.4 [3–4.1	4.2 ± 1.8 [3.1–5.3]	0.48
SET, emotion attribution	–	2.9 ± 1.4 [1–6]	3.1 ± 1.4 [1–6]	3.1 ± 2.1 [0–6]	0.83
CATS					
CATS, identity discrimination	11.7 ± 0.6 [10–12]	11.2 ± 1.2 [9–12]	9.2 ± 2.3 [5–12]*^$**^**^	10.6 ± 2.1 [5–12]	**< 0.001**
CATS, affect discrimination	11.4 ± 0.7 [10–12]	11.3 ± 1.1 [9–12]	9.2 ± 2.2 [2–12]*^$^	10.2 ± 2.0 [5–12]*	**< 0.001**
CATS, affect naming	4.6 ± 1 [2–6]	2.6 ± 1.1 [1–4]*	2.7 ± 1.7 [0–9]*	2.9 ± 2.1 [0–6]*	**< 0.001**
CATS, affect selecting (name emotion target–five faces)	5.5 ± 0.8 [3–6]	3.3 ± 1 [2–5]*	3.4 ± 1.3 [1–6]*	4.1± 1.4 [2–6]*	**< 0.001**
CATS, affect matching (one affect target, five faces)	9 ± 1.9 [5–12]	5.8 ± 0.8 [4–7]*	5.9 ± 2.2 [1–10]*	7.6 ± 2 [4–11]*	**< 0.001**
CATS, affect confrontation (three faces test)	13.7 ± 2.8 [8–19]	10 ± 2.5 [7–16]*	9.5 ± 3 [3–16]*^^^	11.9 ± 2.9 [8–19]	**< 0.001**
BLED					
BLED, picture metaphor	–	2.9 ± 2.4 [0–8]	–	–	NA
BLED, written metaphor	–	3.6 ± 3.6 [0–10]	–	–	NA
BLED, interferences	–	6 ± 1.9 [3–10]	–	–	NA
BLED, requests	–	7.4 ± 2.8 [2–10]	–	–	NA
BLED, humor	–	4 ± 1.8 [1–7]	–	–	NA
BLED, prosody	–	6.7 ± 2.1 [3–10]	–	–	NA
Mood and behavior					
FBI, total	–	22.2 ± 10.3 [11–39]	27.9 ± 12 [10–56]	20.7 ± 12.1 [6–40]	0.14
FBI, A	–	12.6 ± 4.9 [5–20]	16.6 ± 7.1 [0–35]	14.1 ± 8.1 [2–28]	0.17
FBI, B	–	8.1 ± 5.6 [2–17]	10.2 ± 6.9 [0–28]	7 ± 5.2 [0–20]	0.20
NPI, total	–	20.5 ± 19.8 [4–76]	31 ± 21.1 [3–102]	22.1 ± 13.6 [3–46]	0.15
NPI items					
Delusions	–	0.2 ± 0.6 [0–2]	0.5 ± 1.5 [0–8]	0.4 ± 1.4 [0–6]	0.96
Hallucinations	–	0	0.3 ± 1.5 [0–8]	0.4 ± 1.8 [0–8]	0.19
Agitation	–	0.9 ± 1.3 [0–4]	1.9 ± 3.2 [0–12]	3.1 ± 3 [0–9]	0.38
Depression	–	2.3 ± 4 [0–12]	1 ± 2.1 [0–9]	2.4 ± 3 [0–12]	0.31
Anxiety	–	0.7 ± 1.1 [0–3]	1.1 ± 2.8 [0–12]	3 ± 3.3 [0–12]	0.07
Euphoria	–	0.8 ± 1.3 [0–4]	1.9 ± 3.1 [0–12]	0.8 ± 1.8 [0–6]	0.34
Apathy	–	2.8 ± 3.7 [0–12]	6.4 ± 4.1 [0–12]	5.3 ± 4.8 [0–12]	0.33
Disinhibition	–	0.8 ± 1.1 [0–3]	4.3 ± 4.8 [0–12]	2.6 ± 3.5 [0–12]	0.08
Irritability	–	1.5 ± 1.9 [0–6]	2.6 ± 3.6 [0–12]	2.7 ± 3.7 [0–12]	0.46
Motor disturbance	–	1.3 ± 2 [0–6]	2.3 ± 4.5 [0–12]	1.3 ± 3.1 [0–12]	0.49
Nightime behaviors	–	0	1.9 ± 2.8 [0–8]	0.9 ± 1.5 [0–4]	0.21
Appetite/eating changes	–	1.7 ± 2.3 [0–6]	5.2 ± 4.3 [0–12]^$§^	2.1 ± 3 [0–8]	**0.001**

Values are means ± standard deviations [range]. *p *values refer to ANOVA models, Bonferroni-corrected for multiple comparisons, corrected by age, sex and education

Bold is for significant values

*BLED* Batteria sul Linguaggio dell’Emisfero Destro, *CATS* Comprehensive Affect Testing System, *CDT* Clock Drawing Test, *CET* Cognitive Estimation Test, *FAB* Frontal Assessment Battery, *FBI* Frontal Behavioral Inventory, *HC* healthy controls, *MCST* Modified Card Sorting Test, *MMSE* Mini-Mental State Examination, *NPI* Neuropsychiatric Inventory, *RAVLT* Rey Auditory Verbal Learning Test, *SET* Story-based Empathy Test, *TMT* Trail Making Test

*Statistically different from HC

^§^Statistically different from bvFTD

^$^Statistically different from sbvFTD

^^^Statistically different from svPPA

bvFTD group had greater dietary changes and poorer performances at problem solving (MCST), left limb ideomotor praxis (Goldenberg’s test), identity discrimination (CATS, face discrimination), and complex visuospatial constructive abilities (Benson’s figure copy) compared to both sbvFTD and svPPA, and at affect discrimination compared to svPPA (CATS, affect discrimination-three faces). Patients with svPPA were more compromised at semantic fluency compared to both sbvFTD and bvFTD groups, also performing poorer at tests of ideational apraxia compared to bvFTD.

Supplementary Table 5 describes the groups of patients in terms of frequency of pathological performances (based on Italian norms) at each neuropsychological test. Of note, no sbvFTD patient had pathological scores at selective attention (attentive matrices) and showed lower frequency of pathological scores at visual naming and semantic fluency compared to svPPA. Compared to sbvFTD and svPPA patients, bvFTD cases showed higher percentages of pathological scores in tests assessing complex visuospatial constructive abilities (Benson’s figure copy), problem solving and perseverations (MCST), and face discrimination.

Qualitatively, 100% of sbvFTD patients failed in test assessing famous face recognition and affect selecting, and more than 80% failed in tests assessing word comprehension, written metaphor, and humor comprehension subtests of the BLED. On the other hand, no one (0%) failed in tests assessing set-shifting (TMB B, B-A), ideomotor apraxia with the imitation of non-meaning gestures (Goldenberg’ test), and affect discrimination.

### Voxel-based morphometry

**Fig. 3:**
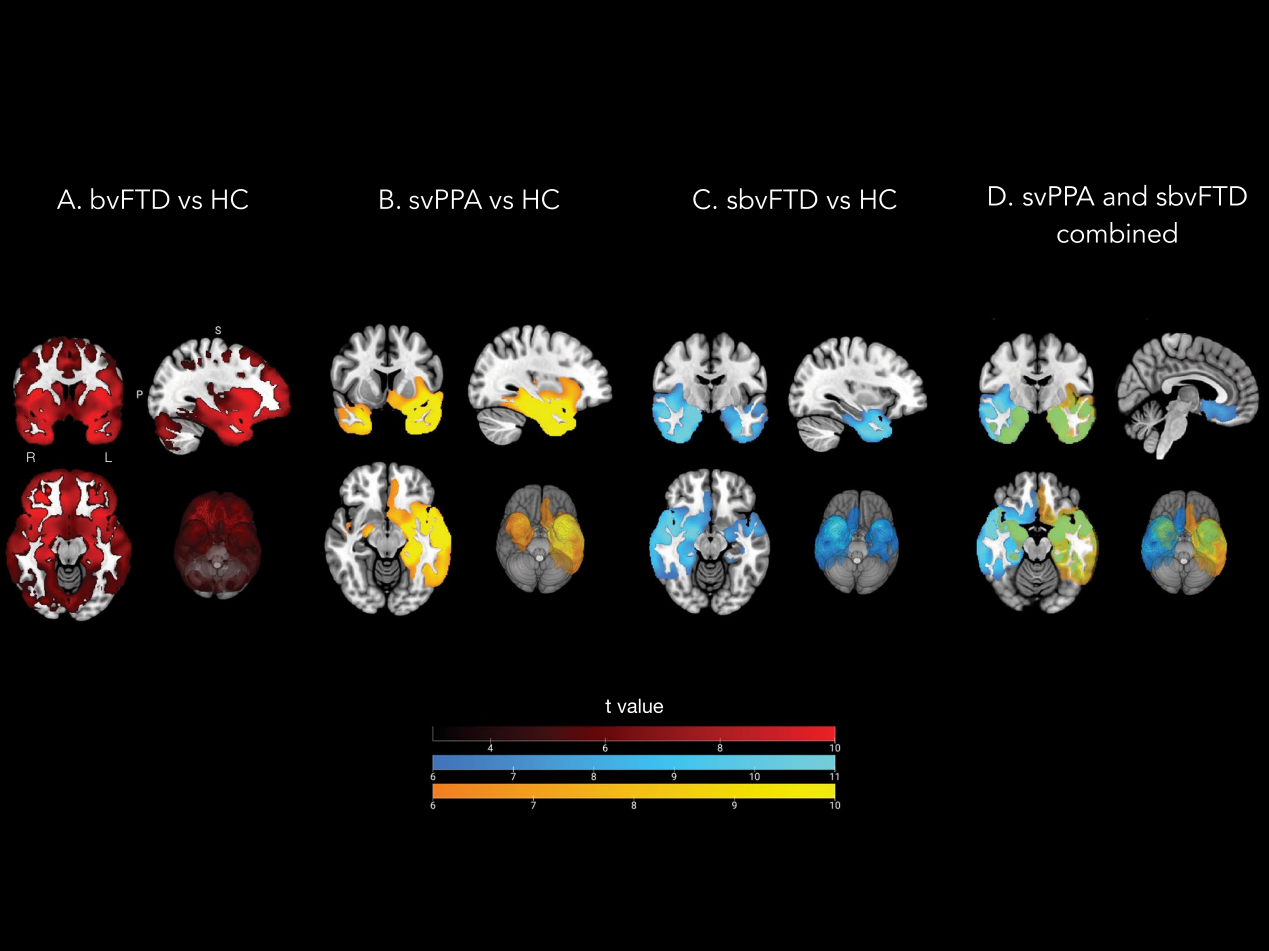
Patterns of gray matter atrophy in patients with behavioral variant frontotemporal dementia (bvFTD), semantic variant primary progressive aphasia (svPPA), and semantic behavioral frontotemporal dementia (sbvFTD). Results of voxel-based morphometry analysis showing regions of significant GM atrophy in bvFTD patients compared to healthy controls (HC) (**A**), svPPA compared to HC (**B**), and sbvFTD compared to HC (**C**). Composite image showing svPPA and sbvFTD patterns of atrophy, combined with green areas showing regions of overlapping volume loss (**D**). Significant clusters are overlaid on sections of the Montreal Neurologic Institute standard brain. Analyses were corrected for age, sex, and total intracranial volume. Statistical threshold for significance was *p* < 0.05, family-wise error corrected for multiple comparisons. *bvFTD* behavioral variant frontotemporal dementia, *sbvFTD* semantic behavioral variant frontotemporal dementia, *svPPA* semantic variant primary progressive aphasia

**Fig. 4:**
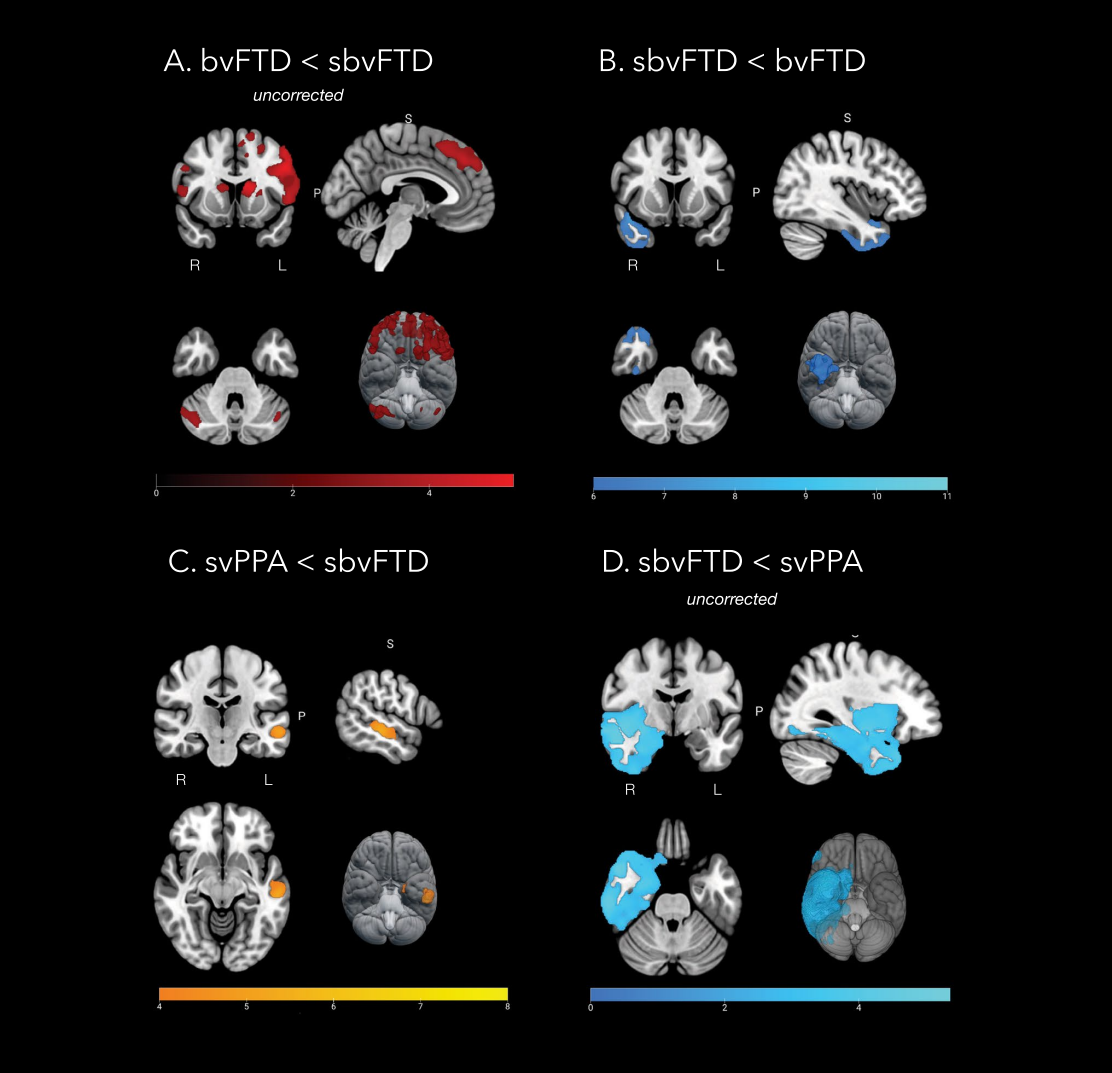
Patterns of gray matter atrophy in patients with semantic behavioral variant frontotemporal dementia (sbvFTD) compared to behavioral variant frontotemporal dementia (bvFTD) and semantic variant primary progressive aphasia (svPPA). Results of voxel-based morphometry showing regions of significant GM atrophy in bvFTD compared to sbvFTD (**A**), sbvFTD compared to bvFTD (**B**), svPPA compared to sbvFTD (**C**), sbvFTD compared to svPPA (**D**). Significant clusters are overlaid on sections of the Montreal Neurologic Institute standard brain. Analyses were corrected for age, sex, and total intracranial volume. Statistical threshold for significance was *p* < 0.05, family-wise error corrected for multiple comparisons. For uncorrected results, threshold of significance was *p* < 0.001. *bvFTD* behavioral variant frontotemporal dementia, *sbvFTD* semantic behavioral variant frontotemporal dementia, *svPPA* semantic variant primary progressive aphasia

As shown by Fig. 3 and Supplementary Table 6, different patterns of atrophy emerged at VBM when comparing group of patients to controls. BvFTD patients (Fig. 3A) showed a pattern of widespread bilateral frontotemporal atrophy, which extended also to occipito-parietal areas and basal ganglia. svPPA patients (Fig. 3B) presented lateralized volume loss in temporal regions, mainly on the left hemisphere, with a relative sparing of frontal cortices but a tendency to spread toward ipsilateral middle frontal gyrus and medial temporal areas. sbvFTD patients (Fig. 3C) presented a mixed phenotype of bilateral ATL atrophy, mainly on the right side with some extension to the ipsilateral cingulum, fusiform, and supramarginal gyrus. Evaluating the patterns of atrophy observed in svPPA and sbvFTD patients, as compared with healthy controls (Fig. 3D), an almost specular pattern of atrophy was found, with rightward regions more affected in sbvFTD and leftward areas more atrophic in svPPA. Still, overlapping areas emerge in ATL, bilaterally. When comparing bvFTD and svPPA patients to sbvFTD, the latter resulted more atrophic in an extensive cluster involving the right ATL (Fig. 4, Supplementary Table 6). Compared to sbvFTD, bvFTD were more atrophic in the left precentral and caudate areas, whereas svPPA showed greater atrophy in the left middle temporal gyrus (Fig. 4, Supplementary Table 6).

## Discussion

This study provides a detailed account of clinical, neuropsychological, neuroanatomical, and genetic data of 15 Italian patients affected by sbvFTD, as identified by retrospectively applying Younes diagnostic guidelines [7] to a multicenter cohort of 236 FTLD-related cases. We showed that these criteria can be effectively used in a clinical context to identify FTD patients with characteristic clinical and anatomical involvement of the right temporal lobe, although we observed significant overlap at a single patient level with previous criteria used to define other FTD variants—in particular, bvFTD [1]. We were able to identify earliest and later symptoms in the disease course, suggest “extra-criteria” clinical features, and describe a global cognitive profile using standardized neuropsychological instruments that may aid in the univocal identification of this syndrome, with the important support of a neuroimaging signature consistent with the complex co-existence of behavioral and (extra-)linguistic semantic deficits observed in sbvFTD.

From the careful review of patient and caregiver reports in search for the taxonomy of symptoms elaborated by Younes et al. [7], we observed that although two of the three core features of sbvFTD (i.e., person-specific semantic knowledge loss, mental rigidity and loss of empathy) were mostly present since early stages, loss of empathy was usually reported only later. Right ATL has been already identified as the “home” for circuits of object and person semantic cognition [19–21], while the medial prefrontal cortex hosts the circuits of empathy [22, 23]. We speculate that loss of empathy is developed later because pathology in the right ATL might only tangentially affect prefrontal circuits of the core empathy network in the initial phase of disease [24], having an indirect effect in developing loss of empathy only as the disease spreads. We, therefore, argue that loss of empathy might not be particularly sensitive in early presentations of sbvFTD, supporting the idea that the fulfillment of only two of three criteria might be sufficient for an sbvFTD diagnosis.

Moreover, a significant proportion of patients in our cohort showed an array of “extra-criteria” behavioral features that went from simple anxiety to suspiciousness, irritability, and histrionic-like behavior. Anxiety, somatic complaints, and hallucinations have already been reported in a cohort of Dutch patients diagnosed with right temporal variant FTD [25]. Dysthymia and anxiety have been associated with right temporal hypoperfusion at SPECT in FTD patients [26]. Another case series demonstrated right temporal lateralization describing cases of partial seizures presenting with anxiety and ictal panic [27]. Therefore, as psychiatric symptoms might be easily misinterpreted as presymptomatic traits, these could actually be an early feature of right ATL involvement. A few patients also reported episodic memory loss, but given the retrospective setting of this study, it is difficult to discern whether memory complaints were due to inaccurate caregiver reporting of a semantic deficit or the unfolding of the disease toward hippocampal memory circuits.

For what concerns neuropsychological features, sbvFTD patients showed a consistent sparing of attentive and executive functions. Difficulties at the TMT test at functional MRI have been localized to the dorsolateral frontal cortex [28, 29] and the relative frontal sparing could account for their intact functions, which differentiate them from bvFTD. Furthermore, given that right ATL atrophy seems to spread contralaterally, relatively sparing frontal lobes, it is possible that executive functions will remain intact for long.

Compared to svPPA, sbvFTD patients tend to perform better at visual naming, semantic, and single-word comprehension. Naming and word comprehension are known to be more impaired in patients with more severe left-sided temporal atrophy and a strong link notoriously exists between naming impairments and the left hemisphere [30].

All sbvFTD patients had a pathological result at the famous face recognition test, corroborating the knowledge that the right ATL mediates the processing and recognition of famous faces [19, 21]. Finally, more than 80% administered with the BLED failed in understanding written metaphors and humor. The engagement of right lateral temporal cortex was associated with tasks of metaphor interpretation [31] and patients with lesions in the right hemisphere have shown difficulties in interpretation of phrases or stories [32]. Humor processing, as well, has been demonstrated to activate right frontotemporal areas [33]. Overall, these findings demonstrate the utility of right hemisphere batteries as a screening tool in patients with sbvFTD.

From a neuroimaging perspective, given that the aim of this study was to classify patients based on diagnostic guidelines [7] and no exclusion was performed a priori if atrophy at MRI extended beyond the right ATL, our sbvFTD cohort presents bilateral ATL atrophy, with a substantial right predominance. sbvFTD and svPPA presented an almost specular pattern of atrophy, in line with the theory that these variants can be considered as the two clinico-anatomical extremes of a “semantic dementia spectrum” [19]. These results are in line with a previous study comparing right temporal variant FTD and svPPA patients, showing, in both groups, a specular involvement of both contralateral temporal and ipsilateral orbitofrontal areas [25]. Another work has demonstrated, with advanced subregion segmentation, the presence of early involvement of the right medial temporal lobe in svPPA patients [34]. The bvFTD group diverged radically, presenting widespread bilateral frontotemporal atrophy, which extended to occipito-parietal areas and basal ganglia. An indirect comparison of MRI features between bvFTD and right temporal variant patients had demonstrated a pattern of widespread medial and lateral temporal lobe atrophy greater in the right hemisphere in the latter, and a largely symmetrical bilateral frontotemporal atrophy encompassing orbitofrontal cortex and bilateral frontal poles in bvFTD, corroborating our results [35]. Being disease duration equal among the three phenotypes, bvFTD present with more extensive neuroanatomical damage, possibly indicating a faster disease course.

The three FTD groups presented a homogeneous distribution of genetic variants. sbvFTD genetic cases showed different mutations (*C9orf72*, *MAPT*, *GRN*), implying the presence of diverse pathologies. Of note, the proportion of patients carrying genetic variants was higher in sbvFTD patients compared to svPPA, which could suggest sbvFTD has a stronger genetic component compared to other phenotypes. However, a more extensive cohort of patients will be needed to confirm this trend.

We acknowledge the serious limitation of the relatively small size of our sbvFTD cohort, although this is the first extensive report of Italian sbvFTD cases. Another important limitation lies in the cross-sectional design of this study, as well as its retrospective nature that limits history collection. Indeed, a prospective questionnaire-oriented anamnestic collection would provide more complete data. Therefore, as features of sbvFTD start being universally recognized and validated, this approach should be adopted for history collection of patients with a suspected sbvFTD diagnosis. Furthermore, prosopagnosia was not consistently tested in the bvFTD and svPPA groups, possibly limiting its detection [25]. Previous studies have reported prosopagnosia in both bvFTD and svPPA cases, but at consistently reduced rates compared to what we have observed in our sbvFTD cohort [36, 37].

In conclusion, the characterization of this novel entity is fundamental to raise awareness among clinicians to facilitate early diagnosis, to tailor cognitive rehabilitation, and to carefully advise caregivers, as the symptomatology experienced by those patients could widely differ from the classic symptoms experienced in other FTD variants.

### Supplementary Information

Below is the link to the electronic supplementary material.

Supplementary file1 (DOCX 81 kb)

## Data Availability

The dataset used and analyzed during the current study will be made available by the corresponding author upon request to qualified researchers (i.e., affiliated to a university or research institution/hospital).
