# Supplementary material for: Clinical and neuroanatomical characterization of the semantic behavioral variant of frontotemporal dementia in a multicenter Italian cohort
Source: J Neurol. 2024 Apr 10;271(7):4203–15. doi: 10.1007/s00415-024-12338-9 (PMC11233398; doi:10.1007/s00415-024-12338-9)
Supplement: Supplementary file 1 — Supplementary file1 (DOCX 81 kb) [file 415_2024_12338_MOESM1_ESM.docx]

**SUPPLEMENTARY MATERIAL**

**MATERIALS AND METHODS**

**Genetic analysis**

The presence of GGGGCC hexanucleotide expansion in the first intron of the *C9orf72* gene was assessed using fluorescent amplicon-length analysis and a repeat-primed PCR assay.[1] A cutoff of ≥30 repeats combined with a typical sawtooth pattern was considered pathologic. In addition, *GRN*, *MAPT*, *TARDBP*, *SOD1*, *FUS*, *TBK1*, *TREM2*, *OPTN*, and *VCP* genes were analyzed by next-generation sequencing and their mutations were confirmed by standard Sanger sequencing.[1]

**Neuropsychological evaluation**

Global cognitive functioning was tested with the MMSE[2] and the frontal assessment battery (FAB);[3] long and short term verbal memory with the Rey Auditory Verbal Learning Test (RAVLT)[4] and the digit span forward,[5] respectively; long and short term spatial memory with the recall and recognition of the Benson’s figure[6] and the spatial span forward;[5] executive functions with the digit span backward,[7] Raven’s colored progressive matrices,[8] Trail making test A and B (TMT A, B),[9] attentive matrices[10] and the Modified Card Sorting Task;[11] social cognition with the Story-based Empathy Task (SET),[12] and the Comprehensive Affect Testing System (CATS);[13] language with the token test,[14] Pyramids and Palm Trees test (PPT),[15] CaGi naming and single-word comprehension test,[16] and with the semantic and phonemic fluency tests;[17] visuospatial abilities with the copy of the Benson’s figure,[6] and the Clock Drawing Test;[18] praxis was tested with orofacial and limb ideational and ideomotor apraxia tests,[10] and with the Goldenberg’s test;[19] the presence of behavioral disturbances with the neuropsychiatric inventory (NPI),[20] and the frontal behavioral inventory (FBI)[21]administered to patients’ caregivers. Healthy controls were administered with the same neuropsychological assessment except for frontal assessment battery (FAB), clock drawing test (CDT), CaGi naming and single-word comprehension test, PPT, praxis tests, cognitive estimation task (CET), story-based empathy test (SET), Right Hemisphere Language Battery (Batteria sul Linguaggio dell’Emisfero Destro-BLED), and frontal behavioral inventory (FBI).

**Statistical analysis**

Normal distribution assumption was checked by means of Q-Q plot and Shapiro-Wilk and Kolmogorov-Smirnov tests. Sociodemographic measures were compared between groups using analysis of variance (ANOVA) models, followed by post hoc pairwise comparisons. Clinical and neuropsychological measures were compared using age-, sex– and education-adjusted analysis of variance (ANCOVA) models, followed by post hoc pairwise comparisons, Bonferroni-corrected for multiple comparisons. Sex and frequencies of pathological tests were computed with a chi-squared test. The threshold of significance was set at p <0.05. SPSS Statistics 22.0 software was used.

VBM group comparisons were tested using ANCOVA models in SPM12, adjusting for total intracranial volume, age and sex. Results were assessed at p <0.05, family-wise error (FWE)–corrected for multiple comparisons.

**TABLES**

**Supplementary Table 1. Percentage of patients that underwent each neuropsychological test.**

|  | **HC** | **sbvFTD** | **bvFTD** | **svPPA** |
| --- | --- | --- | --- | --- |
| N (%) | 40 | 15 | 63 | 25 |
| MMSE | 39 (98%) | 15 (100%) | 60 (95%) | 24 (96%) |
| FAB | - | 15 (100%) | 54 (85%) | 22 (88%) |
| RAVLT, immediate recall | 39 (98%) | 14 (94%) | 56 (85%) | 17 (68%) |
| RAVLT, delayed recall | 39 (98%) | 14 (94%) | 56 (85%) | 17 (68%) |
| Benson figure, recall | 27 (68%) | 12 (80%) | 51 (81%) | 21 (84%) |
| Benson figure, recognition | 27 (68%) | 12 (80%) | 51 (81%) | 21 (84%) |
| Digit span, forward | 39 (98%) | 15 (100%) | 59 (93%) | 24 (96%) |
| Spatial span, forward | 38 (95%) | 14 (94%) | 54 (85%) | 21 (84%) |
| Benson figure, copy | 27 (68%) | 12 (80%) | 51 (81%) | 21 (84%) |
| CDT | - | 14 (94%) | 60 (95%) | 21 (84%) |
| Raven’s colored progressive matrices | 39 (98%) | 14 (94%) | 52 (82%) | 21 (84%) |
| Digit span, backward | 38 (95%) | 14 (94%) | 54 (85%) | 21 (84%) |
| MCST, categories | 39 (98%) | 10 (67%) | 39 (62%) | 15 (90%) |
| MCST, perseverations | 39 (98%) | 10 (67%) | 39 (62%) | 15 (90%) |
| TMT, A | 37 (93%) | 13 (87%) | 50 (79%) | 21 (84%) |
| TMT, B | 37 (93%) | 13 (87%) | 50 (79%) | 21 (84%) |
| TMT, BA | 37 (93%) | 13 (87%) | 50 (79%) | 21 (84%) |
| Attentive matrices | 38 (95%) | 15 (100%) | 57 (90%) | 24 (96%) |
| Token test | 39 (98%) | 14 (94%) | 50 (79%) | 20 (80%) |
| CaGi, visual naming | - | 14 (94%) | 13 (21%) | 20 (80%) |
| CaGi, single-word comprehension | - | 14 (94%) | 9 (10%) | 21 (84%) |
| Phonemic fluency | 35 (88%) | 3 (20%) | 11 (17%) | 8 (32%) |
| Semantic fluency | 35 (88%) | 3 (20%) | 13 (21%) | 10 (40%) |
| Pyramids and Palm Trees test | - | 13 (87%) | - | 19 (76%) |
| Orofacial apraxia, ideomotor | - | 11 (74%) | 49 (77%) | 19 (76%) |
| Orofacial apraxia, ideational | - | 6 (40%) | 38 (60%) | 16 (64%) |
| Limb apraxia, ideational right | - | 6 (40%) | 34 (54%) | 15 (90%) |
| Limb apraxia, ideational left | - | 6 (40%) | 31 (49%) | 15 (90%) |
| Limb apraxia, ideomotor right | - | 12 (80%) | 52 (82%) | 20 (80%) |
| Limb apraxia, ideomotor left | - | 12 (80%) | 42 (66%) | 18 (72%) |
| Goldenberg’s test, right | - | 12 (80%) | 45 (71%) | 19 (76%) |
| Goldenberg’s test, left | - | 12 (80%) | 45 (71%) | 19 (76%) |
| CET | - | 10 (67%) | 10 (16%) | - |
| Famous face recognition test | - | 6 (40%) | - | - |
| Benton face recognition test | - | 6 (40%) | - | - |
| *SET* |  |  |  |  |
| SET, global score | - | 12 (80%) | 41 (65%) | 15 (90%) |
| SET, intention attribution | - | 12 (80%) | 41 (65%) | 15 (90%) |
| SET, causal interference | - | 12 (80%) | 41 (65%) | 15 (90%) |
| SET, emotion attribution | - | 12 (80%) | 41 (65%) | 15 (90%) |
| *CATS* |  |  |  |  |
| CATS, Identity discrimination | 37 (93%) | 12 (80%) | 48 (76%) | 16 (64%) |
| CATS, Affect discrimination | 37 (93%) | 12 (80%) | 48 (76%) | 16 (64%) |
| CATS, Affect naming | 37 (93%) | 12 (80%) | 48 (76%) | 16 (64%) |
| CATS, Affect selecting (name emotion target-five faces) | 37 (93%) | 12 (80%) | 48 (76%) | 16 (64%) |
| CATS, Affect matching (one affect target, five faces) | 37 (93%) | 12 (80%) | 48 (76%) | 16 (64%) |
| CATS, Affect confrontation (three faces test) | 37 (93%) | 12 (80%) | 48 (76%) | 16 (64%) |
| *BLED* |  |  |  |  |
| BLED, Picture metaphor | - | 12 (80%) | - | - |
| BLED, Written metaphor | - | 2 (13%) | - | - |
| BLED, Interferences | - | 12 (80%) | - | - |
| BLED, Requests | - | 12 (80%) | - | - |
| BLED, Humor | - | 12 (80%) | - | - |
| BLED, Prosody | - | 12 (80%) | - | - |
| Mood and Behavior |  |  |  |  |
| FBI, total | - | 11 (74%) | 48 (76%) | 18 (72%) |
| FBI, A | - | 11 (74%) | 48 (76%) | 18 (72%) |
| FBI, B | - | 11 (74%) | 48 (76%) | 18 (72%) |
| NPI, total | - | 11 (74%) | 46 (73%) | 21 (84%) |
| *NPI items* |  |  |  |  |
| Delusions | - | 11 (74%) | 46 (73%) | 21 (84%) |
| Hallucinations | - | 11 (74%) | 46 (73%) | 21 (84%) |
| Agitation | - | 11 (74%) | 46 (73%) | 21 (84%) |
| Depression | - | 11 (74%) | 46 (73%) | 21 (84%) |
| Anxiety | - | 11 (74%) | 46 (73%) | 21 (84%) |
| Euphoria | - | 11 (74%) | 46 (73%) | 21 (84%) |
| Apathy | - | 11 (74%) | 46 (73%) | 21 (84%) |
| Disinhibition | - | 11 (74%) | 46 (73%) | 21 (84%) |
| Irritability | - | 11 (74%) | 46 (73%) | 21 (84%) |
| Motor disturbance | - | 11 (74%) | 46 (73%) | 21 (84%) |
| Nightime behaviors | - | 11 (74%) | 46 (73%) | 21 (84%) |
| Appetite/eating changes | - | 11 (74%) | 46 (73%) | 21 (84%) |

**Supplementary Table 2. MRI acquisition parameters**

|  | Philips Medical System Intera 3T scan | | |
| --- | --- | --- | --- |
|  | T2-weighted | Sagittal 3D FLAIR | 3D high resolution T1-weighted turbo field echo |
| **Repetition time (msec)** | 5500 | 4800 | 7 |
| **Echo time (msec)** | 247 | 270 | 3.2 |
| **Inversion time 1-2 (msec)** | 2550 | 1650 | 1000 |
| **Echo train lenght** | 173 | 167 | 240 |
| **Acquisition time (min)** | 3.45 | 6.15 | 8.53 |
| **Section thickness (mm)** | 1 | 1 | 1 |
| **No. of sections** | 192 | 192 | 204 |
| **Pixel size (mm)** | 1x1 | 1x1 | 1x1 |
| **Matrix** | 256x256 | 256x256 | 256x256 |
| **Field of view (mm^2^)** | 256x256 | 256x256 | 256x256 |

Abbreviations: FLAIR= fluid-attenuated inversion recovery; MRI= magnetic resonance imaging; msec= millisecond; mm= millimeter; No= number; sec=second

**Supplementary Table 3. Details of genetic variants.**

| **Subject** | **Clinical diagnosis** | **Gene** | **Nucleotide change** | **ClinVar Classification** |
| --- | --- | --- | --- | --- |
| **1** | bvFTD | GRN | c.813_816del | P |
| **2** | sbvFTD (Subject 5) | GRN | c.1297C>T | B/LB |
| **3** | bvFTD | GRN | c.264+7G>A | LB |
| **4** | bvFTD | C9orf72 | c.-45+163GGGGCC[>24] | P |
| **5** | bvFTD | GRN | c.960_961del | LP |
| **6** | bvFTD | GRN | c.813_816del | P |
| **7** | bvFTD | GRN | c.99C>A | LB |
| **8** | bvFTD | C9orf72 | c.-45+163GGGGCC[>24] | P |
| **9** | sbvFTD (Subject 12) | C9orf72 | c.-45+163GGGGCC[>24] | P |
| **10** | bvFTD | C9orf72 | c.-45+163GGGGCC[>24] | P |
| **11** | bvFTD | TREM2 | c.482+2T>C | P |
| **12** | svPPA | MAPT | c.1008G>C | P |
| **13** | bvFTD | C9orf72 | c.-45+163GGGGCC[>24] | P |
| **14** | bvFTD | GRN | c.813_816delCACT | P |
| **15** | bvFTD | GRN | c.813_816delCACT | P |
| **16** | bvFTD | GRN | c.709-2A>T | P |
| **17** | bvFTD | GRN | c.2T>C | P |
| **18** | bvFTD | GRN | c.813_816delCACT | P |
| **19** | bvFTD | C9orf72 | c.-45+163GGGGCC[>24] | P |
| **20** | bvFTD | GRN | c.234_235delAG | P |
| **21** | bvFTD | GRN | c.415T>C | VUS |
| **22** | sbvFTD (Subject 11) | MAPT | c.298A>G | P [22] |
| **23** | bvFTD | FUS | c.177C>T | VUS * |

ClinVar Classification: B= Benign; LB= Likely Benign; P=Pathogenic; VUS= Variant of Uncertain Significance**.** The variants were classified according to the guideline of ACMG. *= Exon 3 heterozygous mutation that could result in altered splicing; in silico analyses (MutationTaster, HSF, NetGene2) have suggested a probable pathogenic role of this variant.

**Supplementary Table 4. First three symptoms developed by sbvFTD patients**

|  | **First symptom** | **Second symptom** | **Third symptom** |
| --- | --- | --- | --- |
| **Subject 1** | Episodic memory loss | Lack of judgement and dysexecutive | Complex compulsions and rigid thought process |
| **Subject 2** | Person-specific semantic knowledge loss | Loss of empathy, other behavioral derangements (histrionic-like behavior, irritability) | Words and objects semantic loss |
| **Subject 3** | Simple repetitive behaviors, hoarding or obsessions | Complex compulsions and rigid thought process | Loss of empathy |
| **Subject 4** | Words and objects semantic loss | Complex compulsions and rigid thought process | Person-specific semantic knowledge loss |
| **Subject 5** | Apathy/inertia | Person-specific semantic knowledge loss | Simple repetitive behaviors, hoarding or obsessions |
| **Subject 6** | Episodic memory loss, words and objects semantic loss | Person-specific semantic knowledge loss | Complex compulsions and rigid thought process |
| **Subject 7** | Words and objects semantic loss | Apathy/inertia, complex compulsions and rigid thought process | Person-specific semantic knowledge loss |
| **Subject 8** | Person-specific semantic knowledge loss | Apathy/inertia | Complex compulsions and rigid thought process, other behavioral derangements (irritability) |
| **Subject 9** | Person-specific semantic knowledge loss | Complex compulsions and rigid thought process, hyperorality or dietary changes | Other behavioral derangements (suspiciousness, anxiety) |
| **Subject 10** | Person-specific semantic knowledge loss | Apathy/inertia | Words and objects semantic loss |
| **Subject 11** | Apathy /inertia | Words and objects semantic loss, episodic memory loss | Person-specific semantic knowledge loss |
| **Subject 12** | Simple repetitive behaviors, hoarding, obsessions, loss of empathy | Words and objects semantic loss | Other behavioral derangements (anxiety) |
| **Subject 13** | Words and objects semantic loss | Loss of empathy | Complex compulsions and rigid thought process |
| **Subject 14** | Words and objects semantic loss, apathy/inertia | Simple repetitive behaviors, hoarding, obsessions | Complex compulsions and rigid thought process, hyperorality or dietary changes |
| **Subject 15** | Apathy/inertia | Words and objects semantic loss | Person-specific semantic knowledge loss |

**Supplementary Table 5. Frequency of pathological scores at neuropsychological testing in sbvFTD, bvFTD and svPPA patients**

|  | **sbvFTD** | **bvFTD** | **svPPA** | **p** |
| --- | --- | --- | --- | --- |
| N | 15 | 63 | 25 | - |
| **Memory** | | | | |
| RAVLT, immediate recall (N) | 43% (14) | 55% (55) | 57% (14) | 0.67 |
| RAVLT, delayed recall (N) | 43% (14) | 61% (49) | 45% (11) | 0.63 |
| Benson’s figure, recall (N) | 58% (12) | 80% (51) | 62% (21) | 0.14 |
| Benson’s figure, recognition (N) | 42% (12) | 59% (51) | 48% (21) | 0.46 |
| Digit span, forward (N) | 7% (15) | 17% (59) | 22% (23) | 0.47 |
| Spatial span, forward (N) | 14% (14) | 40% (52) | 14% (21) **§** | **0.03** |
| **Visuospatial abilities** | | | | |
| Benson’s figure, copy (N) | 8% (12) | 52% (50) $^ | 24% (21) | **0.01** |
| CDT (N) | 50% (14) | 65% (60) | 57% (21) | 0.72 |
| **Executive functions** | | | | |
| Raven’s colored progressive matrices (N) | 7% (13) | 33% (52) | 19% (21) | 0.11 |
| Digit span, backward (N) | 8% (13) | 24% (51) | 18% (17) | 0.43 |
| MCST, categories (N) | 30% (10) | 67% (39) $^ | 13% (15) | **0.001** |
| MCST, perseverations (N) | 30% (10) | 76% (38) $^ | 27% (15) | **0.001** |
| TMT, A (N) | 15% (13) | 22% (50) | 20% (20) | 0.87 |
| TMT, B (N) | 0% (13) | 16% (31) | 7% (14) | 0.27 |
| TMT, BA (N) | 0% (13) | 19% (31) | 15% (13) | 0.24 |
| Attentive matrices (N) | 0% (15) **§ ^** | 28% (57) | 30% (23) | **0.05** |
| **Language** | | | | |
| Token test | 29% (14) | 33% (49) | 45% (20) | 0.53 |
| CaGi, visual naming | 64% (14) **^** | - | 100% (19) | **0.008** |
| CaGi, single-word comprehension | 83% (12) | - | 86% (21) | 1.00 |
| Phonemic fluency | 40% (15) | 51% (57) | 42% (19) | 0.66 |
| Semantic fluency | 50% (14)  **^** | 60% (57) | 94% (18) | **0.01** |
| Pyramids and Palm Trees test | 67% (12) | - | 79% (19) | 0.68 |
| **Praxis** | | | | |
| Orofacial apraxia, ideomotor | 57% (7) | 24% (49) | 37% (19) | 0.51 |
| Orofacial apraxia, ideational | 50% (6) | 37% (38) | 88% (16) **§** | **0.003** |
| Limb apraxia, ideomotor right | 8% (12) | 15% (52) | 5% (20) | 0.44 |
| Limb apraxia, ideomotor left | 0% (12) | 14% (37) | 6% (18) | 0.37 |
| Limb apraxia, ideational right | 67% (6) | 35% (34) | 93% (14) **§** | **0.001** |
| Limb apraxia, ideational left | 67% (6) | 32% (31) | 93% (15) **§** | **<0.001** |
| Goldenberg’s test, right | 0% (12) | 20% (45) | 22% (18) | 0.22 |
| Goldenberg’s test, left | 0% (12) | 16% (45) | 0% (18) | 0.08 |
| **Emotion and social cognition** | | | | |
| CET (cut-off 18) | 60% (10) | 67% (9) | - | 0.57 |
| Benton face recognition test | 50% (6) | - | - | NA |
| Famous face recognition test | 100% (6) | - | - | NA |
| *SET* |  |  |  |  |
| SET, global score | 50% (12) | 39% (41) | 40% (15) | 0.79 |
| SET, intention attribution | 50% (12) | 37% (41) | 31% (13) | 0.59 |
| SET, causal interference | 33% (12) | 27% (35) | 20% (15) | 0.74 |
| SET, emotion attribution | 42% (12) | 34% (41) | 47% (15) | 0.67 |
| *CATS* |  |  |  |  |
| CATS, Face discrimination | 17% (12) | 67% (48) **$ ^** | 31% (16) | **0.002** |
| CATS, Affect discrimination | 0% (12) | 31% (48) | 19% (16) | 0.07 |
| CATS, Affect naming | 50% (12) | 64% (33) | 53% (15) | 0.97 |
| CATS, Affect selecting (name emotion target-five faces) | 100% (12) | 96% (46) | 94% (16) | 0.70 |
| CATS, Affect matching (one affect target, five faces) | 8% (12) | 20% (46) | 6% (16) | 0.34 |
| CATS, Affect discrimination (three faces test) | 58% (12) | 58% (45) | 31% (16) | 0.17 |
| *BLED* |  |  |  |  |
| BLED, Picture metaphor | 50% (12) | - | - | - |
| BLED, Written metaphor | 83% (12) | - | - | - |
| BLED, Interferences | 0% (12) | - | - | - |
| BLED, Requests | 58% (12) | - | - | - |
| BLED, Humor | 83% (12) | - | - | - |
| BLED, Prosody | 50% (12) | - | - | - |

Values are percentages (number). P values refer to Chi-square results. Symbols: §= statistically different from bvFTD; $= statistically different from sbvFTD; ^= statistically different from svPPA. Abbreviations: BLED =Batteria sul Linguaggio dell’Emisfero Destro; CATS= Comprehensive Affect Testing System; CDT = Clock drawing test; CET = Cognitive Estimation Test; FAB = Frontal Assessment Battery; MCST = Modified Card Sorting Test; MMSE = Mini Mental State Examination; NA=not available; RAVLT = Rey Auditory Verbal Learning Test; SET= Story-based Empathy Test; TMT = Trail Making Test.

**Supplementary Table 6. Results of Voxel-Based Morphometry.**

Regions where GM loss was more severe in FTD compared with HC and between groups of patients. Regions presented in table survived a p<0.05 FWE-corrected at cluster level.

| **sbvFTD<HC** |  | | | | | |
| --- | --- | --- | --- | --- | --- | --- |
| Anatomic regions (BA) | Side | Cluster size | MNI coordinates | | | T values |
|  |  |  | x | y | z |  |
| Temporal Pole Sup (38) | R | 84546 | 33 | 15 | -28 | 13.22 |
| Cingulum Mid (24) | R | 208 | 3 | 2 | 30 | 5.40 |
| Fusiform (19) | R | 94 | 28 | -78 | -10 | 5.11 |
| Supramarginal (40) | R | 97 | 64 | -30 | 42 | 4.90 |
| Frontal Mid (46) | R | 73 | 45 | 44 | 4 | 4.80 |
| **svPPA<HC** |  |  |  |  |  |  |
| Temporal Pole Sup (38) | L | 55520 | -24 | 10 | -33 | 17.69 |
| Temporal Pole Mid (38) | R | 19718 | 22 | 14 | -34 | 10.86 |
| Angular (39) | L | 271 | -45 | -54 | 38 | 6.12 |
| Occipital Inf (19) | R | 241 | 45 | -76 | -15 | 5.48 |
| Frontal Mid (9) | L | 528 | -26 | 28 | 38 | 5.46 |
| Cerebellum Crus II | R | 238 | 21 | -82 | -34 | 5.15 |
| Frontal Sup (9) | L | 96 | -15 | 48 | 38 | 5.13 |
| Cingulum Mid (23) | L | 266 | -2 | -30 | 36 | 5.04 |
| Parietal Inf (40) | L | 111 | -33 | -40 | 45 | 5.03 |
| Frontal Mid (10) | L | 32 | -32 | 51 | 2 | 5.00 |
| Frontal Mid (10) | L | 121 | -27 | 39 | 28 | 4.99 |
| Cingulum Mid (23) | L | 51 | -4 | -21 | 46 | 4.98 |
| Cingulum Post (23) | L | 83 | -2 | -45 | 21 | 4.94 |
| Cingulum Mid (23) | L | 46 | -2 | -4 | 33 | 4.85 |
| **bvFTD<HC** |  |  |  |  |  |  |
| Insula (13) | R | 249749 | 39 | 20 | 2 | 12.05 |
| Occipital Mid (18) | L | 58 | -21 | -90 | 15 | 5.61 |
| Cerebellum IX | L | 72 | -12 | -40 | -46 | 5.33 |
| Cerebellum IX | R | 36 | 12 | -40 | -48 | 5.11 |
| Parietal Sup (7) | L | 29 | -24 | -58 | 62 | 5.04 |
| Parietal Sup (7) | L | 49 | -20 | -58 | 62 | 4.96 |
| Parietal Sup (7) | L | 29 | -26 | -48 | 60 | 4.88 |
| **bvFTD<sbvFTD** |  |  |  |  |  |  |
| Precentral (6) | L | 511 | -46 | 9 | 32 | 5.24 |
| Caudate | L | 297 | -12 | 8 | 16 | 5.11 |
| **svPPA<sbvFTD** |  |  |  |  |  |  |
| Temporal Mid (21) | L | 581 | -56 | -26 | -8 | 5.07 |
| **sbvFTD<bvFTD** |  |  | x | y | z |  |
| Temporal Pole Sup (38) | R | 17259 | 45 | 10 | -18 | 7.43 |
| Temporal Inf (20) | L | 409 | -38 | -9 | -34 | 5.50 |
| Temporal Pole Sup (38) | L | 224 | -44 | 8 | -24 | 5.15 |
| **sbvFTD<svPPA** |  |  |  |  |  |  |
| Temporal Pole Sup (38) | R | 17092 | 45 | 10 | -15 | 6.79 |
| Fusiform (37) | R | 55 | 58 | -60 | 0 | 4.77 |
| Hippocampus | R | 28 | 36 | -16 | -14 | 4.71 |

**REFERENCES**

1. Spinelli, E.G., et al., *Structural MRI Signatures in Genetic Presentations of the Frontotemporal Dementia/Motor Neuron Disease Spectrum.* Neurology, 2021. **97**(16): p. e1594-e1607.

2. Folstein, M.F., S.E. Folstein, and P.R. McHugh, *"Mini-mental state". A practical method for grading the cognitive state of patients for the clinician.* J Psychiatr Res, 1975. **12**(3): p. 189-98.

3. Appollonio, I., et al., *The Frontal Assessment Battery (FAB): normative values in an Italian population sample.* Neurol Sci, 2005. **26**(2): p. 108-16.

4. Carlesimo, G.A., C. Caltagirone, and G. Gainotti, *The Mental Deterioration Battery: normative data, diagnostic reliability and qualitative analyses of cognitive impairment. The Group for the Standardization of the Mental Deterioration Battery.* Eur Neurol, 1996. **36**(6): p. 378-84.

5. Orsini, A., et al., *Verbal and spatial immediate memory span: normative data from 1355 adults and 1112 children.* Ital J Neurol Sci, 1987. **8**(6): p. 539-48.

6. Possin, K.L., et al., *Distinct neuroanatomical substrates and cognitive mechanisms of figure copy performance in Alzheimer's disease and behavioral variant frontotemporal dementia.* Neuropsychologia, 2011. **49**(1): p. 43-8.

7. Monaco, M., et al., *Forward and backward span for verbal and visuo-spatial data: standardization and normative data from an Italian adult population.* Neurol Sci, 2013. **34**(5): p. 749-54.

8. Basso, A., E. Capitani, and M. Laiacona, *Raven's coloured progressive matrices: normative values on 305 adult normal controls.* Funct Neurol, 1987. **2**(2): p. 189-94.

9. Giovagnoli, A.R., et al., *Trail making test: normative values from 287 normal adult controls.* Ital J Neurol Sci, 1996. **17**(4): p. 305-9.

10. Spinnler, H., Tognoni, G., *[Italian standardization and classification of Neuropsychological tests. The Italian Group on the Neuropsychological Study of Aging].* Ital J Neurol Sci, 1987. **Suppl 8**: p. 1-120.

11. Caffarra, P., et al., *Modified Card Sorting Test: normative data.* J Clin Exp Neuropsychol, 2004. **26**(2): p. 246-50.

12. Dodich, A., et al., *A novel task assessing intention and emotion attribution: Italian standardization and normative data of the Story-based Empathy Task.* Neurol Sci, 2015. **36**(10): p. 1907-12.

13. Froming K, L.M., Ekman P, *The comprehensive affect testing system.* Psychology Software, Inc; Gainesville, FL: 2006.

14. De Renzi, E. and L.A. Vignolo, *The token test: A sensitive test to detect receptive disturbances in aphasics.* Brain, 1962. **85**: p. 665-78.

15. Gamboz, N., et al., *Normative data for the Pyramids and Palm Trees Test in the elderly Italian population.* Neurol Sci, 2009. **30**(6): p. 453-8.

16. Catricalà, E., et al., *An Italian battery for the assessment of semantic memory disorders.* Neurol Sci, 2013. **34**(6): p. 985-93.

17. Novelli G, L.M., Papagno C, Vallar G, Capitani E, Cappa SF, *Three clinical tests to research and rate the lexical performance of normal subjects.* Arch Psicol Neurol Psichiatr., 1986. **47**: p. 477-506.

18. Manos, P.J., *Ten-point clock test sensitivity for Alzheimer's disease in patients with MMSE scores greater than 23.* Int J Geriatr Psychiatry, 1999. **14**(6): p. 454-8.

19. Goldenberg, G., *Neuropsychological assessment and treatment of disorders of voluntary movement*, in *Handbook of clinical neuropsychology*, K. Halligan, & Marshall Editor. 2003.

20. Cummings, J.L., et al., *The Neuropsychiatric Inventory: comprehensive assessment of psychopathology in dementia.* Neurology, 1994. **44**(12): p. 2308-14.

21. Alberici, A., et al., *The Frontal Behavioural Inventory (Italian version) differentiates frontotemporal lobar degeneration variants from Alzheimer's disease.* Neurol Sci, 2007. **28**(2): p. 80-6.

22. Iovino, M., et al., *The novel MAPT mutation K298E: mechanisms of mutant tau toxicity, brain pathology and tau expression in induced fibroblast-derived neurons.* Acta Neuropathol, 2014. **127**(2): p. 283-95.
